# Experimental Evolution of a Bacteriophage Virus Reveals the Trajectory of Adaptation across a Fecundity/Longevity Trade-Off

**DOI:** 10.1371/journal.pone.0046322

**Published:** 2012-10-12

**Authors:** Richard H. Heineman, Sam P. Brown

**Affiliations:** 1 Section of Integrative Biology, University of Texas at Austin, Austin, Texas, United States of America; 2 Centre for Infection, Immunity and Evolution, University of Edinburgh, Edinburgh, United Kingdom; University of York, United Kingdom

## Abstract

Life history theory attempts to account for how organisms lead their lives, balancing the conflicting demands of reproduction and survival. Here, we track the genomic and phenotypic evolution of the bacteriophage virus T7 across a postulated fecundity/longevity constraint. We adapted T7 to a challenging survival environment (6M urea). Our evolved strain displayed a significant improvement in propagule survival, coupled with a significant *loss* of fecundity (reduced growth rate on host cells). However, the increased resistance to urea did not generalise to increased resistance against temperature stress, highlighting that propagule durability is environment dependent. Previous comparative studies predicted that changes in propagule resistance would be mediated by changes in capsid proteins or gene deletions. In contrast, we found that point mutations in internal core protein genes (*6.7* and *16*) were responsible for the increased urea resistance of our evolved strain. Prior to the emergence of the *6.7* and *16* mutations, a distinct set of 5-point mutations peaked at over 20% prevalence before attenuating, suggestive of negative epistatic interactions during adaptation. Our results illustrate that parasites can adapt to specific transmission environments, and that this adaptation can impose costs on the subsequent ability to exploit host cells, potentially constraining durable parasites to lower virulence.

## Introduction

Models based on natural selection make a number of simplifying assumptions in order to draw general conclusions. Most generally, they assume certain traits to be constrained via trade-offs while allowing others to evolve freely. In the absence of constraints, such models would predict a world filled with ‘Darwinian Demons’, organisms that reproduce at a ferocious rate, and yet never die [1]. While it is possible to relax any assumption regarding constraints, in practice this must be done sparingly to preserve the usefulness of the model. Empirical tests are therefore important for identifying which life history trade-offs are most likely to shape evolution.

The principal route to documenting biological constraints is to look for patterns in comparative data – for instance, do species that live longer tend to reproduce more slowly [2]? A recent comparative analysis of bacteriophage virus life histories concluded that this was the case. De Paepe and Taddei [3] reported a 10-fold difference in propagule stability (in a constant and benign environment) across 16 bacteriophages (viruses) of *E. coli*, and further pointed to a significant cost associated with increasing propagule durability. They found that viruses with more durable propagules tended to also be slower to reproduce on *E. coli* host cells, with typically lower burst sizes (number of viral progeny released on the death of a host cell), highlighting a potential fecundity/longevity trade-off in phages.

Viruses are transmitted from host to host as a metabolically inert propagule (a virion), featuring a protein capsule (capsid) encasing a tightly-coiled genome. Transmission depends on the integrity of a protein casing (the capsid), so denaturants such as high temperatures [4] or urea [5] greatly accelerate viral decay. However, it is not well known how evolutionary adaptation to resist one of these selective pressures generalizes even to other relatively similar denaturants.

Durability as a life history trait may be even more complicated by nonlinear phage decay. In animal viruses [4] and phages [6], viral decay in response to heat follows a 2-component inactivation curve, with rapid early decay followed by slower decay of an isogenic thermally resistant population. This subpopulation might have a major effect on the epidemiology and selective pressures acting on viruses under harsh conditions. How general is this nonlinearity across other environmental stresses? Does this alternative phenotype persist after selection for increased durability? Is the adapted phage phenotypically similar to the original resistant population, which might suggest that adaptation largely involved more phages taking on the resistant form?

To answer these questions and test the hypothesized trade-off between durability and growth rate experimentally, we exposed the dsDNA lytic bacteriophage T7 to a stressful environment of 6 M urea. Gupta et al. [5] demonstrated that survival of T7 in 6 M urea is readily improvable via selection. However, they did not explore the possible trade-off between propagule durability and growth rate or explore the genetic basis of adaptation at the population level. Here, we measure correlated changes in other aspects of pathogen life history and examine the genetic basis of evolution. Finally, we discuss the epidemiological and life-history implications of a trade-off between propagule durability and the rate of host exploitation.

## Methods

### Selection for resistance to environmental stress

Our experimental protocol centred on a modified batch-culture protocol, with T7 grown on high density *E. coli* IJ1133 [*E. coli* K-12, F- Δ*lacX74 thi* Δ(*mcrC*-*mrr*)102::Tn*10*], which was also used for all assays. Our ancestral T7 line, T7_A_, was pre-adapted to the host strain and the high-density growth conditions (T7_Hi_ from [7]). During adaptation to urea, the passaging was modified to alternate growth in high density *E. coli* with a survival challenge in 6 M urea. In the growth phase, *E. coli* was grown from a frozen stock (20% glycerol/80% LB, stored at −80°C) to a concentration of 1–2×10^8^ cells/ml in 10 ml of LB (37°C, 200 RPM in shaking water bath). Between 500 and 10^5^ phages were added (roughly ½ the phages that survived the previous urea treatment) and these phages were allowed to replicate until the cell culture had entirely lysed (less than 1 hr), at which point the lysate was chloroformed to kill cells, spun down and filtered (0.2 µm) to remove host cells and cellular debris, and stored on ice. The initial lysate was mutagenised with N-methyl-N'-nitro-N-nitrosoguanidine at 0.5 µg/mL to generate diversity.

The phage culture was then subjected to survival selection. 200 ul of phage in LB was added to 1800 ul of 10 mM Tris-HCl (pH 7.5 at 22°C in a 200 RPM water bath) to a final concentration of 6 M urea. Samples were taken during urea exposure at regular intervals, diluted and stored on ice before plating and plaque counting. As propagule durability improved during the adaptation process, the period of exposure to urea increased from 4–5 min to 20–24 min. Dialysis in 10 mM Tris-HCl (pH 7.5) was used to remove urea before starting next growth phase. After 11 rounds of urea exposure and regrowth, the adaptation resulted in T7_E_.

### Life-history measurements

Both ancestral and evolved strains were assayed for growth rate on *E. coli* and survival over 15 minutes in 6 M urea. Growth rate was measured under conditions similar to those used in the growth phase of adaptation except that phages were not allowed to grow beyond 10^7^ phage/ml (see [7]). Growth is measured in doublings/hour, calculated as [log_2_(*N_t_*/*N*_0_)]/*t*, where *N_t_* is the number of phages in the flask at time *t* hours.

Survival in urea was determined by linear regression of log-transformed titers measured at 1,2,3,4, and 5 minutes of exposure, and expressed as halvings/min (assuming a first order inactivation process). Exponential decay was approximately constant over this time, while longer exposure to urea resulted in significantly slower decay rate. For some assays of T7_A_ and T7_E_, survival was also measured from 10 to 15 minutes. All urea survival assays were done at 24°C, but conditions were otherwise identical to those used during adaptation. Survival (or inactivation) was also assayed for filtered cultures in LB without urea under a range of temperature regimes (37°C over 50 days and 63°C over 5 hours). In order to ensure independence of data points and avoid contamination, each time point in the heat assays was sampled from a different tube.

### Sequencing

In order to characterize the genetic changes underlying the period of adaptation to environmental urea, T7_E_ was fully sequenced, as were all sites in the recombinant phage (see below) that differed between evolved and ancestral strains. This sequencing was performed from PCR products using an ABI3100 analyzer and DNA Star Lasergene Seqman software (version 5.05). We also sequenced the entire genome of T7 at three points along the adaptation using 454 pyrosequencing to reveal polymorphism. Genomic DNA was isolated from lysates grown from the growth phase of passages 3, 8 and 12. Indels and sequence changes present at less than 2.5% were ignored to prevent false positives for mutations. Wild-type T7 (GenBank accession number V01146, [8]) was used as a reference sequence to define the locations of changes.

### Recombinant strain engineering

In order to identify the phenotypic impact of mutations, T7_A_ and T7_E_ were cut with restriction enzymes and re-ligated [9] to generate T7_+g*6.7*+g*16*_, a genome with only the coding changes (in genes *6.7* and *16*) that spread during adaptation. These were then transfected into competent IJ1126 cells [*E. coli* K-12, F- *recC*22 *sbcA*5 *endA* Gal- *thi supL* Δ(*mcrC*-*mrr*)102:Tn*10*].

## Results

T7 was adapted under conditions selecting for both survival and growth rate. The phenotypic correlates of this adaptation were assayed, and the genetic basis of survival identified.

### Evolution of urea durability and associated reproductive cost

**Figure 1:**
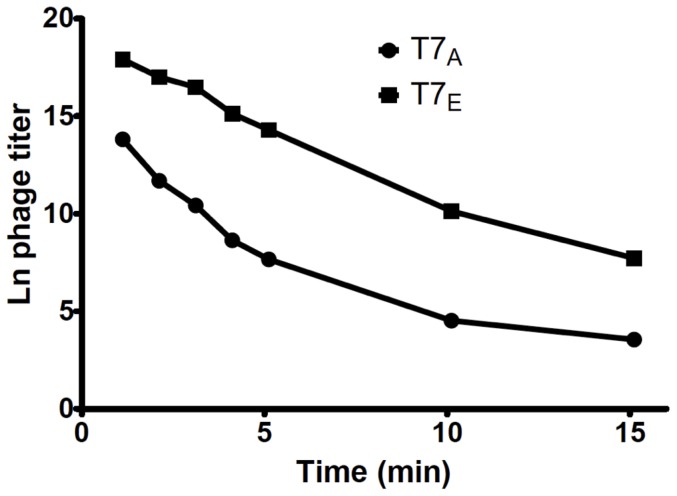
Representative decay curves in 6 M urea for ancestral phage T7_A_ and evolved phage T7_E_.

T7 rapidly adapts to a selective challenge from environmental urea. The original phage (T7_A_) has an initial decay rate of 2.35 halvings/min, while after only 11 passages through urea, the evolved phage (T7_E_) decays at 1.38 halvings/min (Figure 1, Table 1; 2-tailed t-test, *p*<0.0006). In addition to the selected increase in propagule durability, we also found a correlated response in growth rate of the phage on high density *E. coli*, with mean growth rate *decreasing* during experimental evolution from a growth rate of .80 doubling/min to a growth rate of 0.77 db/min (Figure 1, Table 1; 2-tailed t-test, *p*<0.05), despite continued selection for growth. Our experimental results therefore reveal a trade-off; the evolved strain is more durable in urea, yet has a reduced growth rate.

**Table 1:** Phenotypic traits of phage lines with standard errors (computed from observations) and number of assays.

		T7_A_			T7_E_			T7_+g*6.7*+g*16*_	
Fitness (db/min)	0.8	±	0.0051 (4)	0.77	±	0.0086 (5)			
Halvings/min (urea, 1–5 min)	2.35	±	0.088 (3)	1.38	±	0.092 (4)	1.51	±	0.055 (4)
Halvings/min (urea, 10–15 min)	0.17	±	0.11 (2)	0.7	±	0.03 (3)			
Halvings/min (63°C, 0–1 hr)	0.13	±	0.007 (2)	0.14	±	0.008 (2)			
Halvings/min (63°C, 1.5–5 hr)	0.047	±	0.0001 (2)	0.056	±	0.0098 (2)			
Halvings/day (37°C)	0.43	±	0.003 (2)	0.41	±	0.03 (2)			

### Survival in response to other environmental conditions

We then measured the rate of decay of our evolved and ancestral phage lines under distinct environmental challenges, to examine whether resistance to urea generalises to resistance to other environmental stressors. Long-term measurements of decay in relatively benign conditions (no urea, 37°C) showed no significant difference in the rates of decay of evolved and ancestral phages (Table 1, 2-tailed t-test, p<0.61). The phages also do not decay differently under conditions of significant thermal stress (63°C, Table 1, ANOVA, *p*<0.19).

We also attempted to adapt T7_A_ to hot (63°C) water. Despite imposing 99.99% mortality over 3 overnight passages, we found no apparent response to selection (data not shown).

### Variable rates of decay

For both T7_A_ and T7_E_, the constant decay model provided an excellent fit over the first 5 minutes, with R^2^ values largely around 0.98–0.99 and never lower than 0.92. However, decay rate was significantly slower between minutes 10–15 (T7_A_ from 2.35 to 0.17 halvings/min, *p*<0.004; T7_E_ from 1.38 to 0.70 halvings/min, *p*<0.006). Similarly, in the 63°C experiments, we found that for both T7_A_ and T7_E_ the phages appear to die faster within the first hour than they do from 1.5 to 5 hours (ANOVA, p<0.0004). There was no such pattern observed following exposure to 37°C. As decay assays used lysates grown from isolates, it is unlikely that this consistent nonlinearity is a result of genetic variance in the population.

### Genetic basis of a trade-off

**Figure 2:**
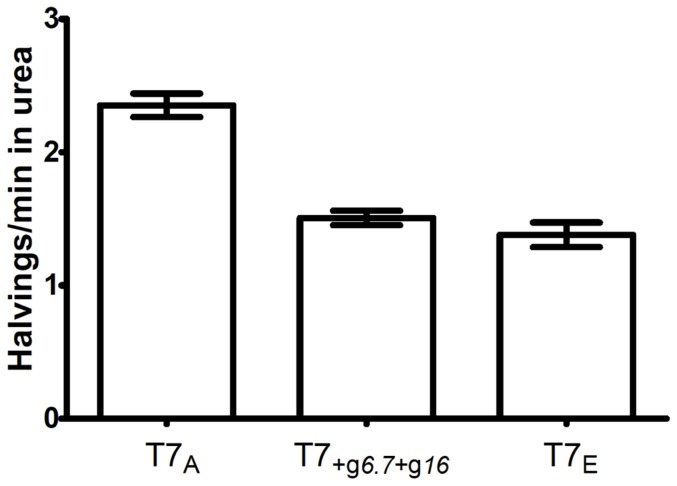
Survival of ancestral, evolved, and recombinant phages in 6 M urea. Error bars indicate ±1 standard error.

Only 5 genetic changes spread during adaptation, all of which were point mutations and only two of which (in genes *6.7* and *16*) were coding (Table 2). In order to confirm that only coding changes were responsible for survival evolution, we created a recombinant strain with only coding changes and assayed for survival in urea. The resultant phage (T7_+g*6.7*+g*16*_) had a death rate of 1.51 halvings/min, greatly decreased from that of T7_A_ (2.35 halvings/min, p<0.004 by 2-tailed t-test) and similar to that of T7_E_ (1.38 halvings/min, p<0.29 by 2-tailed t-test). This suggests that the increased durability of the evolved strain is largely due to coding changes (Figure 2).

**Table 2:** Genetic changes during adaptation, relative to wild-type T7.

Nucleotide	Gene	Change	Gene function	T7_A_	T7_E_	G3	G8	G12
1257–2736	*0.3–0.7* fusion,	Deletion	Various^a^	+	+	+	+	+
	*0.4–0.6* deletion							
15094	*5*	G->T A248S	DNA Polymerase	+	+	0.745	0.928	0.925
24088	*10B*	G->A E375K	Minor coat protein	+	+	+	+	+
30861	*16*	A->C Q89H	Internal core protein	+	+	+	+	+
30945	*16*	T->G N117K	Internal core protein	+	+	+	+	+
32860	*16*	G->A G756S	Internal core protein	+	+	+	+	+
34975	*17*	A->G T118A	Tail fiber	+	+	+	+	+
36492	*17.5*	T->G I50S	Suspected holin	+	+	+	+	+
189	Terminal repeat	C->T			−	−	0.051	0.028
3456	1	C->T R96C	RNA polymerase		−	−	−	0.029
4886	1	G->A G572G	RNA polymerase		−	−	−	0.037
7435	1.3	C->T P321S	DNA ligase		−	−	0.107	0.054
8108	1.6	C->T A68V	Non-essential, unknown		−	−	0.545	0.484
11152	3.5	C->T D149D	Lysozyme, regulation		−	−	0.123	0.085
12565	*4A*	G->A G334D	Helicase		−	−	−	0.026
19032	*6.7*	G->A E57K	Adsorption, virion head protein	+	−	0.85	0.955	
19923	7.7	G->A V26M	Non-essential, unknown		−	−	−	0.025
20240	7.7	A->G (ochre to amber)	Non-essential, unknown		−	0.288	0.047	0.058
21027	8	C->T S263F	Head-tail connector		−	−	−	0.036
24537	11	T->C Y104H	Tail protein		−	0.047	−	−
27135	12	C->T T765I	Tail protein		−	−	0.034	0.039
27574	13	A->G M90V	Internal head protein		−	0.204	0.045	0.081
29770	15	G->A K482K	Internal core protein		−	0.257	0.058	0.05
30719	*16*	C->A P42H	Internal core protein		+	0.029	0.693	0.705
35083^a^	*17*	C->T L154L	Tail fiber		+	0.228	0.092	0.138
35385^a^	*17*	G->A Q254Q	Tail fiber		+	−	0.036	0.043
36093	17	C->T L490L	Tail fiber		−	−	0.039	−
39566^a^	After *19.5*	C->A			+	0.306	0.076	0.056

a indicates noncoding change.

+indicates mutation present at greater than 97.5%.

−indicates mutation present at lower than 2.5%.

### Polymorphisms in an adapting population

**Figure 3:**
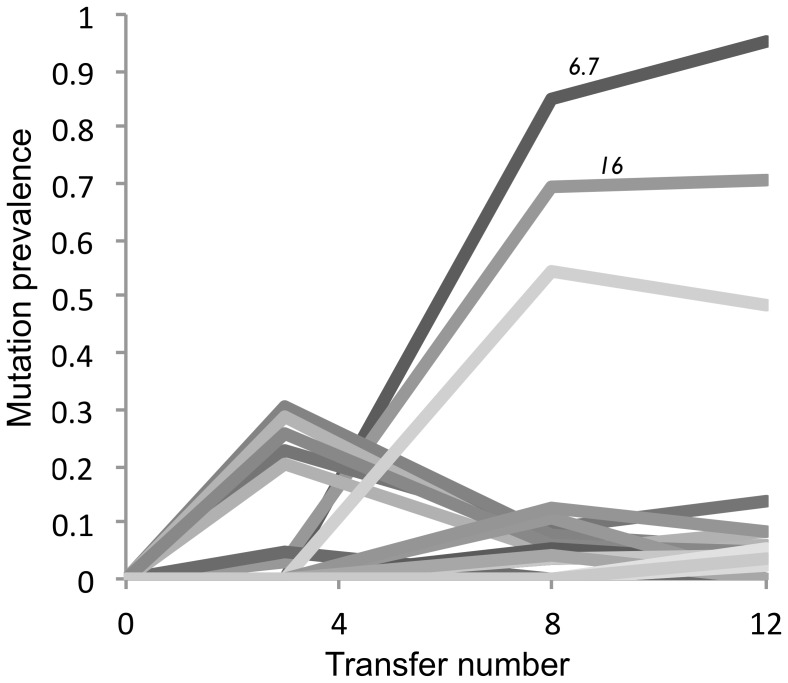
Mutation prevalence over genomic evolution. Ancestral clone T7_A_ is the reference strain; evolved clone T7_E_ was sampled from the population at transfer number 12. Functionally significant mutations to genes *6.7* and *16* are highlighted. Full data presented in Table 2.

There were a number of other changes present in the adapting population, suggesting a more complicated route to adaptation than might be surmised by the small number of mutations required for the final trade-off. Most interestingly, a number of mutations, including a coding change in gene *13*, had spread to relatively high frequency (greater than 20%) by the end of the third growth period but were then lost entirely (Figure 3, Table 2). The mutations responsible for the trade-off in the final population were present only at very low rates at this point. After this initial burst of changes, most mutations not found in T7_E_ were present only at low frequency, with the exception of a change in gene *1.6*. No mutations were fixed over the course of adaptation, despite enormous increases in virion survivability during adaptation.

## Discussion

### Propagule trade-offs and comparative data

Our results in urea experimentally confirm the existence of a phenotypic trade-off between viral survival and reproduction (Figure 1), that had previously been inferred through a comparative analysis of the life-history characteristics of 16 coliphages [3]. However, our experimentally observed trade-off is far less constraining than that inferred from the comparative data. Taking only the 12 lytic double-stranded DNA viruses from De Paepe & Taddei's [3] dataset, we can infer that a doubling of propagule survival can be bought for an approximate halving of viral growth rate (growth rate re-calculated as Ln(burst size)/latency period). In contrast, our evolved doubling of propagule durability in T7 was bought for only a ∼3% reduction in growth rate (Figure 1); why the discrepancy in the magnitude of the trade-off?

The use of comparative data to determine life-history trade-offs is a valuable first step to identifying evolutionary constraints, but it is difficult to know the cause of the correlation when we do not know the selective pressures organisms have faced [2]. Our results illustrate that the T7 virus can readily adapt to improve propagule survival against at least some environmental stressors, but suggest that different environmental challenges may entail very different trade-off landscapes. Thus the relatively weak trade-off that we observed between fecundity and longevity in urea may be stronger in other environments. When we measured durability under conditions more closely resembling those used in de Paepe and Taddei [3], we found that the evolved and ancestral strain had little difference in durability, in keeping with the minimal change in growth rate (Table 1). The relative cheapness of adaptation to survive in urea may not apply to heat resistance, consistent with our inability to register any immediate response to selection on heat resistance.

Even more contradictory comparative results on propagule durability and longevity were reported by [10], who observed a *positive* correlation between virulence and propagule durability across a comparative survey of viral and bacterial pathogens of humans. One possible explanation for this discrepancy lies in the evolution in certain species of a discrete spore forming environmental dispersal stage, distinct from the within-host propagule responsible for cell-to-cell dispersal. By elaborating distinct dispersal stages to face more perilous environmental challenges, the trade-off between environmental survival and within-host (or patch) proliferation may be effectively decoupled. A further and more general explanation for conflicting comparative results is that the ranking of ‘propagule quality’ or durability is likely to be highly sensitive to the environmental assay used to measure durability, as highlighted by our measurements in heat and urea. These results imply that propagule survival (like pathogen growth, and consequent virulence) is a complex and multi-dimensional trait, which cannot be readily reduced to simple two-dimensional trade-offs. To begin to unravel the complexities of survival, we now turn to the molecular mechanisms that caused the phenotypic shift towards greater resistance to urea.

### Mechanisms of a trade-off

How has the virus changed to become more resistant to urea, and yet slower growing on *E. coli*? De Paepe and Taddei's [3] comparative analysis identified two key statistical predictors of a virus's position along a fecundity/longevity tradeoff: the density of DNA packaging and the volume of the capsid. Small capsids with a high density of DNA were associated with the production of many fragile propagules (e.g. R17, T7), whereas big, loosely packed capsids were more costly yet tougher (e.g. PRD1, P4). This suggested that T7 adaptation to urea would involve changes in gene *10* (coding for the capsid protein), or release of packaging pressure via large deletion mutations. In line with these predictions, an earlier adaptation of T7 to survive in urea resulted in a large deletion [5]. Interestingly, segmented genomes in an RNA virus also survived better than unsegmented ones, suggesting a wide range of possibilities for decreasing pressure from genetic material, although no tradeoff was found in this case [11].

There were no deletions in our adaptation, however. This lack of deletions may be because our ancestral strain had already undergone a deletion mutation during an earlier adaptation to lab passaging conditions [9]. This adaptation involved minimal selection for durability, and a similar deletion shortens the generation time of T7 under some conditions (unpublished data), suggesting the primary benefit of the change is in infection. However, this does not rule out a pleiotropic effect on survival, potentially boosting the durability of our ancestral T7_A_ strain relative to wild type T7.

While the capsid protein was predicted to evolve in response to harsh conditions, phenotypic evolution was instead driven by changes to other virion proteins. Gene *16* encodes an internal core protein involved in genome entry [12]. The change was in the muralytic domain [13], which degrades the cell wall at initiation of infection [14], and which frequently evolves during selection for rapid growth rate [9]. In fact, T7_A_ carries 3 changes in this region. It is possible that the new change compensates for a stability cost of these earlier changes [15]. Gp6.7 is an internal head protein that may act as a plug to stabilize the genome inside the capsid [16]. Thus the gp6.7 change is likely to prevent genome destabilization or even release of the genome from the virion upon exposure to urea, suggesting that the mechanism by which urea kills T7 is in large part genome damage. Gp6.7 is involved in morphogenesis [16]; a change that provides greater stability may also slow virion production.

Urea, like heat, is a protein denaturant [17], and for this reason we suspected that correlated evolution was likely. The independence of survival in these two environments emphasizes that the precise nature of environmental pressures affect how phages will respond on a molecular level.

### Complexity of adaptation

The temporal dynamics of population genetic structure reveals a distinct pattern of two sequential waves of mutant expansion/contraction (Figure 3, Table 2). The first wave is characterised by 5 point mutations that reach or exceed 20% frequency at passage 3, before receding. The second, rising wave is characterised by many of the mutations featured in T7_E_ and in particular, the functionally significant changes to genes *16* and *6.7*. Clonal interference is unlikely to completely explain this pattern, given that each growth phase of the adaptation ended with very high multiplicity of infection, and thus ample potential for recombination of beneficial alleles. Besides, while hitchhiking may have caused the spread of some of the mutations, it is unlikely to have been a general driver of the first wave, as all 5 mutants subsequently decreased in frequency. While some of the changes may have increased in frequency due to bottlenecking during death steps, bottlenecking to 500+ phages is unlikely to have resulted in the spread of so many neutral mutants to such high frequency. We suggest that the sequential pattern is most likely the result of negative epistasis, with the first wave of mutations representing an alternative, inferior pathway to resistance, antagonistic to the second wave.

The lack of fixation of any allele throughout the course of adaptation (Figure 3, Table 2), despite dramatic shifts in phenotype, is surprising. Very many viruses were eliminated during each survival challenge, which should lead to strong selection for even small survival differences. However, this was mitigated by the trade-off with growth rate and the large impact of non-genomic factors on survival, which may frequently be the case with life history traits in nature. It is, of course, also possible and even likely that more time could have resulted in fixation of the changes leading to resistance.

### Propagule survival as a complex and multi-dimensional trait

In addition to a strong environmental sensitivity in both resistance and the evolvability of resistance, we also find temporal variation in decay rates, with decay rate slowing during continued exposure. While some recent studies have assumed that the process of phage inactivation follows a first order kinetic [3], [5], other studies have found similar nonlinear effects in response to degradation at high temperature [4], [6]. We demonstrate that this effect, unlike the evolution of resistance, is conserved between high temperature and urea environmental pressures, although in accordance with other studies linearity was restored at lower temperatures [4]. The non-constancy of phage decay in time as well as across environments points to the coexistence of multiple pathways of degradation (e.g. protein denaturation, nucleotide cleavage) and multiple and interchangeable phenotypic states (e.g. extent of nucleotide hydrogenation or binding to metal ions) characterised by different resistances, as inferred for several viruses [4]–[6]. For example, the bacteriophage ΦX174 exists in two phenotypic forms; one is more thermostable but attaches less well [6]. It is possible that in some viruses phenotypic variation represents a viral bet hedging strategy; a sub-set of genetically identical but more resistant propagules will hedge against the arrival of harsher conditions, much as some seeds that germinate late may increase the multiplicative fitness of a plant by protecting against a bad year [18]. In the case of ΦX174, on the other hand, individual virions respond to harsh conditions by undergoing conformational changes that grant them increased resistance [6].

If an analogous phenotypic plasticity applies to T7, one obvious evolutionary response to selection on durability would be for the phage to produce only more stable forms, or transform to them under even mild conditions. Under our adaptive conditions, these resistant viruses, genetically identical to the entire viral population, made up the vast majority of the viruses transmitted to the next generation. Thus, by far the most important targets of selection were for increased likelihood of entering this subpopulation or for survival rate once in it.

Interestingly, the nongenetic variation persists after selection. In fact, this variation is larger than the difference between evolved and ancestral phages; the ancestral virus decayed more slowly from 10–15 min than the evolved did from 1–5 min (Figure 1, Table 1). The persistence of nonlinear decay suggests that it might be a constraint on the system. Even a small proportion of phages with a higher survival rate would be of disproportionate importance to determining the selection pressures exerted by harsh environments. Given the seemingly chaotic environment that phages are faced with in nature, these “persistor” individuals may greatly affect microbial population dynamics, and persistors in animal viruses may be epidemiologically important, playing a role analogous to bacterial spores.

Within this complex adaptive landscape, our results illustrate that the T7 virus can readily adapt to improve propagule survival against at least some environmental stressors, but that this resistance cannot be generalised to other environmental challenges. The differences between survival in urea and at high temperature suggests that phages, and viruses in general, may face a wide range of survival-based selective pressures, some of which are easily responded to and others which are not. In this way survival may be as complicated as growth rate, which is also subject to diverse and contrasting selective pressures in different environments [7], [19]–[25]. Understanding how and when parasites can adapt to environmental challenges via changes in propagule design will help broaden our understanding of pathogen ecology, evolution and control.

### Parasite life-history constraints and implications for virulence

The implications of a trade-off between viral fecundity and longevity are far-reaching. If similar results hold for animal viruses, a number of medical implications can be considered. First, on a within-host scale, our results imply that viruses can potentially adapt to certain extra-cellular control agents (i.e. components of an immune response, or extra-cellular anti-viral drugs), and furthermore that this adaptation may yield a reduction in the growth rate of the virus on cells in the absence of the control agent – a cost of resistance mediated by propagule design. A key difference from our experimental framework is that the growth environment (within cells) and the survival challenge (among cells) are found within the same patch or host, thus the virus within a multi-cellular host must face a significant extra-cellular survival challenge after every round of intra-cellular replication (as opposed to after several rounds of replication in favourable within-patch conditions, as in our experimental protocol). Brown *et al.* [26] theoretically explored resistance evolution (in an intra-cellular bacterial pathogen) to extra-cellular drug control, and predicted an evolutionary shift towards prolonged intra-cellular growth and consequently a reduced overall growth rate, as part of a ‘refuge’ strategy, guarding against frequent extra-cellular exposure. Our results lend further strength to the prediction of longer and slower intra-cellular growth as a response to extra-cellular control, as a result of both a ‘refuge’ strategy [26] and as a result of a mechanistic constraint of adaptation to enhance inter-cellular propagule survival.

Moving to an epidemiological scale, we can now consider the impact of pathogen adaptation to external transmission environments, i.e. the evolution of *indirect* transmission. Our model system was a bacteriophage virus, but we can again extrapolate to animal viruses if we consider the high-density *E. coli* flask to represent a host (or more generally, a resource patch) and the urea flask to represent the transmission environment. Our results therefore point to a trade-off between propagule survival and the within-host growth rate of the pathogen, which can often be related to virulence (a positive link between within-host growth and host mortality has been observed across a range of pathogens, including viruses [27], bacteria [28] and protozoa [29], and is a basic assumption of many theoretical models of virulence evolution (e.g. [30])). Our results therefore lead to a reversal of the curse of the pharaoh hypothesis (the hypothesis that parasites with long-lived propagules will be highly virulent, [31]–[37]); we predict that by *adapting* to survival in extreme transmission environments, pathogens may instead become less able to rapidly – i.e. virulently – exploit their hosts.
